# Study protocol for SeniorWorkingLife - push and stay mechanisms for labour market participation among older workers

**DOI:** 10.1186/s12889-019-6461-6

**Published:** 2019-01-31

**Authors:** Lars Louis Andersen, Emil Sundstrup

**Affiliations:** National Research Centre for the Working Environment, Lersø Parkalle 105, DK-2100 Copenhagen, Denmark

**Keywords:** Senior worker, Ageing, Occupational health, Public health, Workplace, Sustainable employment

## Abstract

**Background:**

Due to demographic changes across Europe there are strong political interests in maintaining the labour force by prolonging working life, i.e. increasing retirement age. This may pose both challenges and opportunities for societies, workplaces, and individuals. The SeniorWorkingLife (Danish: SeniorArbejdsLiv) project investigates push and stay mechanisms for labour market participation – now and in the future - among older workers (≥50 years).

**Methods:**

In July 2018, 30,000 Danes age 50 or older (18,000 employed, 7000 unemployed, 3000 voluntary early retirements, 2000 disability pensions) were invited to participate, of which 15,721 (52.4%) replied to the entire questionnaire and 17,885 (59.6%) replied at least in part. Baseline data collection was terminated in October 2018. The questionnaire covers 14 domains in relation to push and stay mechanisms for labour market participation: 1) basic information (demographics, employment status etc.), 2) multiple-choice question covering a wide range of push and stay mechanisms, 3) role of the workplace, 4) age-discrimination, 5) personal economy, 6) possibility for voluntary early retirement among employed and unemployed, 7) gradual retirement, 8) competencies and continued education, 9) return-to-work, 10) new technologies at the workplace, 11) job satisfaction and well-being, 12) working environment, 13) lifestyle, 14) health and functional capacity. The project aspires to repeat the survey as a prospective cohort every 2–3 years and to perform longitudinal follow-up in Danish high-quality registers about work and health.

**Discussion:**

The SeniorWorkingLife project will provide important knowledge about push and stay mechanisms for labour market participation among older workers. Push refers to mechanisms that increase the risk of premature exit from the labour marker, e.g. due to poor health, poor working environment, age discrimination, and stay to mechanisms prolonging working life e.g. due to attractive working conditions and a good working environment. The project will also to some degree investigate stuck, pull and jump mechanisms. Collaboration and use of the data for scientific purposes by other researchers are encouraged. Interested researchers should contact the corresponding author.

**Trial Registration:**

Registered as cohort study in ClinicalTrials.gov Identifier NCT03634410 (August 16, 2018).

## Background

Demographic changes in many western societies are resulting in a growing ageing population, which is expected to reduce the relative proportion of working-age citizens [1]. This will put economies and welfare systems under pressure due to reduced income taxes and more extensive use of health care services. To resist this pressure, increased labour market participation among older workers has gained vast political attention in recent years, and many European countries are consequently regulating national pension schemes to increase retirement age. This may pose both challenges and opportunities for societies, workplaces and individuals. For instance, health problems limit the ability to sustain employment for individuals in some groups of society [2, 3]. In relation to withdrawal from the labour market, factors stimulating early withdrawal from the labour market are known as push, pull and jump mechanisms, and factors stimulating late withdrawal are known as stay and stuck mechanisms [4]:

**Push** – early involuntary withdrawal, e.g. being pushed out due to poor health or poor working conditions (e.g. age discrimination, stressed or heavy physical work environment).

**Pull** - early voluntary withdrawal due to attractive retirement schemes and/or norms and conventions.

**Jump** – early voluntary withdrawal triggered by the need to realize potentials, wishes, and needs.

**Stay** – late voluntary retirement, e.g. due to being offered attractive working conditions, such as a fulfilling job, good salary, good relationships with management and colleagues, etc.

**Stuck** - late involuntary retirement, e.g. because the economic situation does not permit one to withdraw or a concern about social isolation.

While pull, jump, and stuck mechanisms are largely related to pension schemes and economy, push and stay factors may be more modifiable on the workplace and individual level and are thus the main target of the present project. To keep a larger proportion of older workers at the labour market, increased knowledge on factors associated with choosing to stay longer, i.e. a voluntary and positive choice (stay mechanisms), and involuntary premature labour market exit, i.e. involuntarily being pushed out (push mechanisms) could help to target and integrate initiatives and stimulate sustainable employability in future years.

The reasons for older workers leaving the labour market are complex and dynamic, but there is no doubt that the working environment plays an important role in achieving a long and healthy working life [5–8]. Hence, a large number of studies have investigated factors of importance for premature exit from the labour market. These studies show, among other things, that there are many risk factors in the working environment for poor health and early retirement from the labour market, e.g. high physical work demands and poor psychosocial working conditions [6, 8–10]. A prospective study of employees from the Helsinki Health Study cohort found that physical workload was among the primary risk factors for all-cause disability pension [9]. In addition, Labriola and co-workers found that approximately 21 and 34% for men and women, respectively, of the disability pensions were attributable to ergonomic work environment exposures. A recent systematic review and meta-analysis on the role of psychosocial, social, and organizational work factors on premature exit from the labour market, reported moderate evidence for the role of low job control, and for the combination of high demands and low control (job strain) as predictors for disability pension [9]. The influence of psychosocial working conditions was further established by Christensen and co-workers showing that decision authority and variation explained 10–15% of the risk for disability pension after the adjustment for ergonomic work factors, age, smoking, and BMI [7].

On the other hand, existing knowledge of what makes older people choose to work after the official retirement age (stay mechanisms) is far more limited [11]. Studies conducted have typically investigated the influence of factors such as economic incentives, health and working conditions (e.g. flexible workplaces, extra days off), while less is known on the importance of the work environment for prolonging working lives [12]. A study from the Netherlands found health, work characteristics, skills and knowledge, social factors, and economic to be important factors for the motivation for working beyond retirement age [13]. Working beyond retirement age have been related to various forms of non-economic motivation, with a further distinction between motives that relate to social relationships at the workplace, or that relate to the content of work [4, 14, 15]. In the Danish cross-sectional study entitled “Work, unemployment and withdrawal”, 75% of older workers reported good relationships with colleagues as a motivation for continuing to work at a high age, while 69% indicated a good relationship with the management as a motivator [4]. An important reason for working beyond retirement also seems to adhere to the content of the work, as 84% ​​in the cross-sectional study reported an interesting and rewarding job as an important motivation to continue working [4].

The SeniorWorkingLife (Danish: SeniorArbejdsLiv) project investigates push and stay mechanisms for labour market participation – now and in the future - among older workers or those who have recently retired. The data collected in 2018 will serve as a baseline of the project, which aspires to repeat the survey as a prospective cohort every 2–3 years and to perform longitudinal follow-up in Danish high-quality registers about work and health.

## Methods

### Study design

The data collection was performed between July and October 2018 and forms the baseline of a prospective study aspiring to do long-term follow-up using Danish national registers and questionnaire surveys every 2–3 years. The baseline survey will also be used for cross-sectional and retrospective analyses. The study is registered as a cohort study in ClinicalTrials.gov (Identifier: NCT03634410). Because the study is not a clinical trial – but a cohort study - the SPIRIT guideline is not applicable and has not been used.

### Study population

In July 2018, 30,000 Danes age 50 or older as of 31st March 2018 (18,000 employed, 7000 unemployed, 3000 on voluntary early retirement, 2000 on disability pension) were invited to participate. A total of 15,721 (52.4%) replied to the entire questionnaire and 17,885 (59.6%) replied at least in part. Statistics Denmark drew a probability sample from the Danish Population Register and sent out a personal link to the questionnaire through a secure Danish mailing system, e-Boks, used to send digital mail from public or private companies to citizens [16]. The system is connected to the Danish social security number and therefore follows individuals from birth to death. Login to e-Boks requires both a personal password and a paper-based code, and the system is highly secure. Consequently, the likelihood of someone else responding to the questionnaire survey is minimal. In case of non-response, two digital reminders were sent within the collection period and participants also received a reminder by telephone. A minority of Danes have opted out of the e-Boks system, and these individuals received the invitation as well as the reminders by postal mail. Baseline data collection was terminated in October 2018.

*Employed individuals* were defined based on three criteria. First, the person should have paid employment at least 20/37 h per week (~ 86.6/160.3 h per month) for at least half of the months during the last year as of March 2018. Second, the person should be employed at least 20/37 during March 2018. As the last criteria, the person should not have received benefits in terms of flex-job, sheltered job, sickness absence or maternity/paternity leave during the first quarter of 2018. Furthermore, the sample was stratified for occupational industry with a 50/50 compromise between the number of employees in each occupational industry in Denmark and an equal proportion in each stratum. This process ensures both representativeness of the sample and statistical power to compare between the strata.

*Unemployed individuals* were defined based on two criteria. First, they should be unemployed during the entire first quarter of 2018. Second, they should not have been unemployed for more than 1 year as of March 2018.

*Voluntary early retirement* (Danish: efterløn) is a special type of pension that only certain people are entitled to. Two criteria should be fulfilled. First, the employee should pay a monthly fee for the scheme, which is co-financed by the state. Second, those born before 1st Jan 1954 were qualified for voluntary early retirement payment at the age of 60 given the duration of the payment was 30 years and the person was available for work. However, the employee could postpone retirement age until the age of 62 and thereby receive a higher voluntary early retirement benefit. The benefit stopped at the Danish state pension age of 65 years and individuals shifted to state pension benefits (Danish: folkepension). Thus, the maximal period was 5 years. For those born later than 1st Jan 1954, the eligible age now is gradually increasing and the maximum period decreasing. For example, for those born between July and December 1955, the eligible age is 62 years (i.e. in 2017), and the benefits stop at 67 years, i.e. still a maximal period of 5 years. However, the maximal period decreases in the years to come. In the present study, we have only invited those who recently went on voluntary early retirement, defined as within the last year as of March 2018.

*Disability pension* (Danish: førtidspension) is only possible for Danish residents with a significant and permanent loss of workability, whether due to a specific disease or otherwise. The municipality decides whether a person is entitled to disability pension. To be qualified for a disability pension, an attempt to increase work ability must have been carried out without success. Normally the person goes through a longer process with the involvement of different departments in the municipality (work-, health-, education-, and social-department) before the disability pension can be granted. In the present study, we have only invited those who recently went on disability pension, defined as within the last year as of March 2018.

### Survey variables

The survey is divided into 14 domains. A summary of the most central questions in each domain is described in the following:

1. Basic information:

- Demographics
- Employment status with four categories 1) employed, 2) unemployed, 3) voluntary early retirement, 4) disability pension.
- Job function category with three categories 1) office work, administration, analysis, IT, 2) working with people, service, care, 3) working with processing, producing or moving things
- Expected retirement age (employed and unemployed) and actual retirement age (voluntary early retirement and disability pension).
- Plans for remaining at the labour market with four categories ranging from ‘staying as long as possible’ to ‘would already have left if possible’.

2) Multiple-choice question covering a wide range of push and stay mechanisms:

- Multiple choice question about reasons for expecting to leave the labour market at the age provided in the reply to this question in domain 1 (employed and unemployed) and actual reasons for leaving the labour market at the age provided in the reply to this question in domain 1 (voluntary early retirement and disability pension). Fifteen response options, e.g. poor health, not feeling well at work, common to leave at a certain age, possibility for voluntary early retirement or pension, economic considerations, wish from a spouse, termination of employment, not being able to work anymore, make room for younger employees, more leisure time. The question was inspired from The Danish Longitudinal Study of Ageing [17].
- Possible reasons for staying longer in the labour market than to the age given in section 1. Fifteen reasons, e.g. if the work was less strenuous, if there were better working hours, longer vacations or more senior days, if there was a higher level of recognition and influence at work, if there were more challenges at work, if there was support from spouse, if health were better, if there were better possibilities for continuing education. The question was inspired from The Danish Longitudinal Study of Ageing [17].
- Expected and actual reason for leaving the labour market, with four categories 1) have/had to leave, 2) own choice, 3) combination of the first two, 4) don’t know.

3) Role of the workplace:

- Whether the workplace has a written senior-policy
- Multiple-choice question about possibilities at the workplace, with 15 response options e.g. senior counseling, reduced working time, flexible working hours, development of competencies, days off, reduced workload and responsibility, better salary, health promotion offers.
- Whether there had been a talk with the workplace about future employment, what came out of this talk and how the talk had been experienced by the employee.

4) Age-discrimination:

- At what age employees are considered as “old” at current (employed) or most recent workplace (unemployed, voluntary early retirement, disability pension).
- Multiple-choice question concerning perceptions about what the management thinks about older workers. Ten categories, with both positive and negative perceptions, e.g. older workers are an important resource, productive, flexible, easy to work with and older workers have outdated skills, think mainly about their pension, and create conflicts.
- Experience of age-discrimination in different situations
- Relative age of nearest leader
- Feeling wanted at the workplace

5) Personal economy:

- Expected (employed, unemployed) and actual economic situation (voluntary early retirement, disability pension) after leaving the labour market compared with when working. The question was inspired from the Work, Unemployment and Early Retirement study [18].
- Concerns about personal economic situation
- Economic counseling
- Actual economic situation

6) Possibility for voluntary early retirement among employed and unemployed:

- Whether the respondent is paying to the ‘voluntary early retirement’ scheme and have a certificate that is ready to use
- Whether the respondent can afford to go on voluntary early retirement
- Whether the respondent want to go on voluntary early retirement

7) Gradual retirement:

- Expectations about (employed, unemployed) or actual use of (voluntary early retirement, disability pension) reduced working time as part of the transition from work to pension
- Possibilities for reduced working time
- Perceived expectations from management in case of reduced working time
- Interest in more or less responsibility at work during the year to come (employed)

8) Competencies and continued education:

- Required experience to handle current/former job
- Changes in demands to competencies during last 2 years in current/former job
- Offers and use of education, retraining courses etc. within the last 2 years in current/former job

9) Return-to-work (unemployed, voluntary early retirement, disability pension):

- Work beside pension (voluntary early retirement or disability pension)
- Reason for working beside pension (voluntary early retirement or disability pension)
- Reasons for unemployment (unemployed) [18]
- Perceptions about age as a reason and barrier for unemployment (unemployed)
- Beliefs about returning to work (unemployed) [18].
- Barriers for returning to work (unemployed) [18].
- Available resources that can help in returning to work [18].
- Desire to have a work (unemployed, voluntary early retirement, disability pension)

10) New technologies at the workplace:

- Introduction of new technologies in work during the last 2 years [19].
- Seven questions (yes/no) about positive and negative aspects of new technology [19].

11) Job satisfaction and well-being:

- Job satisfaction, on a 5-point scale from very satisfied to very dissatisfied [20].
- Life satisfaction, on a 10-point scale from very dissatisfied to very satisfied, from the World Values Survey [21].

12) Working environment:

- Basic information about the work, e.g. weekly working hours, number of employees, working schedule (day, evening, night, changing), position (employee or leader) [22].
- Physical activity at work, with 4 categories ranging from mainly sedentary to heavy and fast work [23].
- Perceived physical and mental exertion at work, scale 0–10, where 0 is not strenuous at all and 10 is maximally strenuous [24].
- Psychosocial working environment from the Working Environment & Health study [22], which is based on the second version of the COPSOQ questionnaire [25] and the Danish Psychosocial Questionnaire [26].

13) Lifestyle:

- Smoking habits, alcohol habits, BMI (bodyweight / height^2^), physical activity during leisure [27]

14) Health and functional capacity:

- General health [28]
- Work Ability Index, Item 1 and 2 [29]
- Accidents at work [30]
- Chronic diseases [31]
- Treatments during the last year
- Musculoskeletal pain intensity and duration [22, 32]
- Work limitations due to musculoskeletal pain [22]
- Mental health and vitality [28]
- Cohen’s Perceived Stress Scale (CPSS) [33]
- Bergen Insomnia Scale [34]
- Work Role Functioning [35]

### Registers

The questionnaire data will be merged with a range of Danish registers about e.g. labour market status, work sector, job group, sickness absence, education, income, health, age, gender. A detailed description in English of the registers is available at Statistics Denmark’s homepage [36]. The survey data will be merged with these registers through the unique social security number assigned to all Danish residents at birth or immigration.

### Non-response

Table 1 shows the response percentages among the different subgroups. Non-response is defined as 100% - percentage replying to the entire questionnaire. Non-response varied across the different subgroups of the study population. Among the four main strata, the response percentage was, from high to low, 66.7% (voluntary early retirement), 56.0% (employed), 45.6% (unemployed) and 27.9% (disability pension). The response percentage was relatively high in all subgroups of employed, but in general higher in subgroups with more seated work than in subgroups with more physical work. Women replied to a slightly higher degree than men. For the age-groups, the younger subgroups of the + 50 years replied to a lesser degree than the older subgroups. Immigrants replied to a lesser degree than those of Danish origin and descendants of immigrants. Those with higher education also responded to a higher degree than those with lower education. There were also slight variations of across the five regions of Denmark (range 50–56%). For income, there was an increasing response with higher income, except for the smaller group with no registered income.

**Table 1:** Number invited and number who replied to the entire questionnaire (percentage in parenthesis) in each subgroup

	Invited	Replied to entire questionnaire. Number (percentage)
Total	30,000	15,721 (52.4%)
1. Employed, total	18,000	9974 (56.0%)
2. Unemployed	7000	3189 (45.6%)
3. Voluntary early retirement	3000	2000 (66.7%)
4. Disability pension	2000	558 (27.9%)
Employed, subgroups		
1. Agriculture, forestry and fishing	474	222 (46.8%)
2. Industry, mineral extraction and utilities	2321	1265 (54.5%)
3. Construction	1350	608 (45.0%)
4. Trade and transport, etc.	2538	1280 (50.4%)
5. Information and communication	1084	622 (57.4%)
6. Finance and insurance	1250	724 (57.9%)
7. Real estate and rental	762	363 (47.6%)
8. Business services	1813	983 (54.2%)
9. Public administration, education and health	5164	3158 (61.2%)
10. Culture, leisure and other services	1244	749 (60.2%)
Sex		
Men	16,238	8117 (50.0%)
Women	13,762	7604 (55.3%)
Age (1st April 2018)		
50–54 years	10,205	4592 (45.0%)
55–59 years	9202	4799 (52.2%)
60–64 years	9396	5621 (59.8%)
65+ years	1197	709 (59.2%)
Origin		
1. Danish	27,711	14,849 (53.6%)
2. Immigrants	2219	833 (37.5%)
3. Descendants of immigrants	70	39 (55.7%)
Highest completed education		
1. Primary school or unknown	6576	2722 (41.4%)
2. Highschool	14,047	7302 (52.0%)
3. Short-term higher education.	1642	935 (56.9%)
4. Medium-term higher education.	4934	3055 (61.9%)
5. Long-term higher education.	2801	1707 (60.9%)
Region of Denmark		
1. North Jutland	3247	1737 (53.5%)
2. Mid Jutland	6370	3535 (55.5%)
3. Southern	6548	3495 (53.4%)
4. Capital	9096	4555 (50.1%)
5. Zealand	4739	2399 (50.6%)
Family income (in 1000 DKr)		
1. No income	1462	655 (44.8%)
2. -150	1696	432 (25.5%)
3. 150–250	6593	2893 (43.9%)
4. 250–350	9675	5325 (55.0%)
5. 350–500	7619	4658 (61.1%)
6. 500+	2955	1758 (59.5%)
Family type		
1. Single, no children living at home	7492	3264 (43.6%)
2. Single, children living at home	1200	524 (43.7%)
3. Couple, no children living at home	15,012	8712 (58.0%)
4. Couple, children living at home	6296	3221 (51.2%)

Table 2 shows reasons for non-response in the study population. There were various reasons for non-response. The most common reason was that people denied replying, did not respond to the reminder phone call, or were registered with a wrong/expired phone number.

**Table 2:** Responders and reasons to not respond

	Number (percentage)
Invited	30,000 (100%)
Responders	
Replied to the entire questionnaire	15,721 (52.4%)
Replied to parts of the questionnaire	2164 (7.2%)
Non-responders, reason	
Denied to reply	2683 (8.9%)
Misc non-response	217 (0.7%)
Language difficulties	112 (0.4%)
Wrong or expired phone number	2976 (9.9%)
Not possible to find phone number	2647 (8.8%)
Not responded to telephone call	3480 (11.6%)
Did not find it relevant	0 (0%)
Not contacted	0 (0%)

### Weights

Due to the different size and response percentage of subgroups, weights will be used in the forthcoming analyses of the study results. The model assisted weights will be based following background variables (where strata are the four types of employment status, i.e. employed, unemployed, voluntary early retirement, and disability pension):

Sex * age group * strata.

Occupational industry * strata.

Highest completed education * strata.

Family income * strata.

Family type * strata.

Origin * strata.

### Planned analyses

The study will – based on the 14 domains above – analyse Push and Stay factors for labour market participation …

1. … in the four main strata (employment, unemployment, voluntary early retirement, disability pension).
2. … in the 10 occupational industries (listed in Table 1).
3. … in subgroups of job function category (from domain 1 above) crossed with educational attainment.
4. … in relation to expected retirement age (employed and unemployed)

The database will be linked with relevant registers as mentioned above, and the project aspires to repeat the survey as a prospective cohort every 2–3 years and to perform longitudinal follow-up in Danish high-quality registers about work and health.

The project will also to a certain extent analyse stuck, pull and jump mechanisms. For example, a poor economic situation can be a ‘stuck’ mechanism, i.e. the individual is forced to stay in the labour market due to economic reasons in spite of poor health.

The project will also benefit from the long history of Danish registers to perform retrospective analyses, e.g. to analyse the influence of previous labour market history/mobility – e.g. number and length of previous employment and periods of unemployment and sickness absence - on the push and stay mechanisms for labour market participation.

## Discussion

The SeniorWorkingLife project investigates push and stay mechanisms for labour market participation, both now and in the future, among older workers or those who have recently retired. The data collected in 2018 will serve as a baseline of the project, which aspires to repeat the survey as a prospective cohort every 2–3 years and to perform longitudinal follow-up in Danish high-quality registers about work and health.

We expect that the results from the study will contribute with research that can qualify the ongoing debate about prolonged working careers and contribute to that more seniors in the future can withdraw from the labour market with dignity when it is necessary to leave early, e.g. due to health reasons, and have increased opportunities at the labour market when desiring to stay longer.

The study has both strengths and limitations. The sample was drawn as a probability sample by Statistics Denmark among all eligible Danish residents age 50 years or older that fitted into the four categories of employed, unemployed, voluntary early retirement, and disability pension. The overall response percentage was satisfactory (i.e. 52.4% replied to the entire questionnaire and 59.6% replied at least in part) and in line with previous large-scale Danish studies investigating work environment and health [24, 37]. However, among those granted a disability pension, the response percentage to the entire questionnaire was only 28%. Regardless of the difficult situation of the persons granted a disability pension - which may partly explain the low response percentage - this is a major limitation from a research perspective in relation to generelizability and interpreting the results from that group. It is likely that the responders are better functioning and have better health than the non-responders, and differences between those granted a disability pension and the three other strata of employment status may be less pronounced than if a higher response percentage in this group was obtained. This may lead to more conservative results. For this and other reasons, statistical weights will be used when comparing across the different subgroups of employment status. Due to the methods used and the overall response percentage, the generalizability is in general high.

## Abbreviations

BMI: Body mass index
COPSOQ: The Copenhagen Psychosocial Questionnaire

## References

[1] Ilmarinen J (2006). The ageing workforce--challenges for occupational health. Occup Med (Lond).

[2] Reeuwijk KG, van Klaveren D, van Rijn RM, Burdorf A, Robroek SJW (2017). The influence of poor health on competing exit routes from paid employment among older workers in 11 European countries. Scand J Work Environ Health.

[3] Leijten FRM, de Wind A, van den Heuvel SG, Ybema JF, van der Beek AJ, Robroek SJW (2015). The influence of chronic health problems and work-related factors on loss of paid employment among older workers. J Epidemiol Community Health.

[4] Goul Andersen J, Jensen PH, Goul AJ (2011). Tilbagetrækning fra arbejdsmarkedet - årsager og effekter.

[5] Poulsen OM. Arbejdsmiljøets betydning for fastholdelse af ældre arbejdstagere. 2017. http://nfa.dk/da/Forskning/Udgivelse?journalId=5bafb358-c476-4717-bc43-c5bd05064f08. Accessed 9 Jan 2019.

[6] Labriola M, Feveile H, Christensen KB, Strøyer J, Lund T (2009). The impact of ergonomic work environment exposures on the risk of disability pension: prospective results from DWECS/DREAM. Ergonomics.

[7] Christensen KB, Feveile H, Labriola M, Lund T (2008). The impact of psychosocial work environment factors on the risk of disability pension in Denmark. Eur J Pub Health.

[8] Sundstrup E, Hansen ÅM, Mortensen EL, Poulsen OM, Clausen T, Rugulies R (2018). Retrospectively assessed physical work environment during working life and risk of sickness absence and labour market exit among older workers. Occup Environ Med.

[9] Knardahl S, Johannessen HA, Sterud T, Härmä M, Rugulies R, Seitsamo J (2017). The contribution from psychological, social, and organizational work factors to risk of disability retirement: a systematic review with meta-analyses. BMC Public Health.

[10] Robroek SJW, Schuring M, Croezen S, Stattin M, Burdorf A (2013). Poor health, unhealthy behaviors, and unfavorable work characteristics influence pathways of exit from paid employment among older workers in Europe: a four year follow-up study. Scand J Work Environ Health.

[11] Wahrendorf M, Akinwale B, Landy R, Matthews K, Blane D (2017). Who in Europe works beyond the state pension age and under which conditions? Results from SHARE. Population Ageing.

[12] Carlstedt AB, Brushammar G, Bjursell C, Nystedt P, Nilsson G (2018). A scoping review of the incentives for a prolonged work life after pensionable age and the importance of “bridge employment”. Work.

[13] Sewdas R, de Wind A, van der Zwaan LGL, van der Borg WE, Steenbeek R, van der Beek AJ (2017). Why older workers work beyond the retirement age: a qualitative study. BMC Public Health.

[14] Clement SL, Andersen JG. LEDIGHED OG INCITAMENTSEFFEKTER: HVAD VED VI? En forskningsoversigt. CCWS; 2006.

[15] Graversen G, Holt LH. Arbejdslivets psykologi. Kbh: Hans Reitzel. 2007.

[16] e-Boks. What is e-Boks? https://www.e-boks.com/danmark/en. Accessed 9 Oct 2018.

[17] VIVE – The Danish Center for Social Science Research (2018). The Danish Longitudinal Study of Ageing (Ældredatabasen).

[18] Andersen JG (2017). Arbejde, ledighed og tilbagetrækning, 2006.

[19] FTF. Welcome to the machine: FTF; 2018. https://fho.dk/blog/2017/04/27/naar-ansatte-efteruddannes-i-ny-teknologi-oeges-kvalitet-og-tilfredshed/. Accessed 29 Jan 2019.

[20] Andersen LL, Fishwick D, Robinson E, Wiezer NM, Mockałło Z, Grosjean V (2017). Job satisfaction is more than a fruit basket, health checks and free exercise: Cross-sectional sectional study among 10,000 wage earners. Scand J Public Health.

[21] Elgar FJ, Davis CG, Wohl MJ, Trites SJ, Zelenski JM, Martin MS (2011). Social capital, health and life satisfaction in 50 countries. Health Place.

[22] National Research Centre for the Working Environment. Working Environment & Health in Denmark 2012-2020. 2018. http://nfa.dk/da/Arbejdsmiljoedata/Arbejdsmiljo-i-Danmark/Arbejdsmiljo-og-helbred-i-Danmark. Accessed 10 Oct 2018.

[23] Hein HO, Suadicani P, Gyntelberg F (1992). Ischaemic heart disease incidence by social class and form of smoking: the Copenhagen male study--17 years’ follow-up. J Intern Med.

[24] Andersen LL, Thorsen SV, Flyvholm M-A, Holtermann A (2018). Long-term sickness absence from combined factors related to physical work demands: prospective cohort study. Eur J Pub Health.

[25] Pejtersen JH, Kristensen TS, Borg V, Bjorner JB (2010). The second version of the Copenhagen psychosocial questionnaire. Scand J Public Health..

[26] Clausen T, DPQ Research Survey (2018). National Research Centre for the working environment.

[27] Calatayud J, Jakobsen MD, Sundstrup E, Casaña J, Andersen LL (2015). Dose-response association between leisure time physical activity and work ability: cross-sectional study among 3000 workers. Scand J Public Health.

[28] White MK, Maher SM, Rizio AA, Bjorner JB (2018). A meta-analytic review of measurement equivalence study findings of the SF-36® and SF-12® health surveys across electronic modes compared to paper administration. Qual Life Res.

[29] Ilmarinen J (2009). Work ability--a comprehensive concept for occupational health research and prevention. Scand J Work Environ Health.

[30] Ajslev JZN, Sundstrup E, Jakobsen MD, Kines P, Dyreborg J, Andersen LL (2018). Is perception of safety climate a relevant predictor for occupational accidents? Prospective cohort study among blue-collar workers. Scand J Work Environ Health.

[31] Sundstrup E, Jakobsen MD, Mortensen OS, Andersen LL (2017). Joint association of multimorbidity and work ability with risk of long-term sickness absence: a prospective cohort study with register follow-up. Scand J Work Environ Health.

[32] Pincus T, Bergman M, Sokka T, Roth J, Swearingen C, Yazici Y (2008). Visual analog scales in formats other than a 10 centimeter horizontal line to assess pain and other clinical data. JRheumatol.

[33] Cohen S, Kamarck T, Mermelstein R (1983). A global measure of perceived stress. J Health Soc Behav.

[34] Pallesen S, Bjorvatn B, Nordhus IH, Sivertsen B, Hjørnevik M, Morin CM (2008). A new scale for measuring insomnia: the Bergen insomnia scale. Percept Mot Skills.

[35] Johansen T, Lund T, Jensen C, Momsen A-MH, Eftedal M, Øyeflaten I (2018). Cross-cultural adaptation of the work role functioning questionnaire 2.0 to Norwegian and Danish. Work.

[36] Statistics Denmark (2018). Documentation of statistics.

[37] Andersen LL, Fallentin N, Thorsen SV, Holtermann A (2016). Physical workload and risk of long-term sickness absence in the general working population and among blue-collar workers: prospective cohort study with register follow-up. Occup Environ Med.

[38] Committee System on Biomedical Research Ethics. Guidelines about Notification etc. of a Biomedical Research Project to the Committee System on Biomedical Research Ethics. 2011. http://www.nvk.dk/~/media/NVK/Dokumenter/Vejledning_Engelsk.pdf. Accessed 26 Nov 2018.

[39] The Danish Data Protection Agency. The Data Protection Act. 2018. https://www.datatilsynet.dk/media/6894/danish-data-protection-act.pdf. Accessed 26 Nov 2018.

